# Liuwei Dihuang Lowers Body Weight and Improves Insulin and Leptin Sensitivity in Obese Rats

**DOI:** 10.1155/2012/847167

**Published:** 2011-09-04

**Authors:** Benjamin Perry, Junzeng Zhang, Changhao Sun, Tarek Saleh, Yanwen Wang

**Affiliations:** ^1^Institute for Nutrisciences and Health, National Research Council Canada, Charlottetown, PE, Canada C1A 4P3; ^2^Department of Biomedical Sciences, University of Prince Edward Island, Charlottetown, PE, Canada C1A 4P3; ^3^Department of Chemistry, University of Prince Edward Island, Charlottetown, PE, Canada C1A 4P3; ^4^Department of Nutrition and Food Hygiene, Harbin Medical University, Heilongjiang, Harbin 150081, China

## Abstract

The present study was aimed at investigating the efficacy and mechanism(s) of action of a Chinese herbal formulation, Liuwei Dihuang (LWDH), as a prospective natural weight-lowering product. Following a 2-week acclimation period, 48 obesity-prone (OP-CD) rats were divided into 4 groups (*n* = 12 each). One group served as a positive control for obesity (OP), while the other 3 were challenged twice daily by oral gavage with total daily dosages of 500, 1500, or 3500 mg/kg BW LWDH, respectively, for 10 weeks. One group (*n* = 12) of obesity-resistant (OR-CD) rats served as the normal control group. All rats were fed the same AIN-93G diet modified to contain 60% energy from fat. The highest LWDH dose significantly reduced body weight during the last 4 weeks of treatment. Food intake was reduced beginning in week 2. The high LWDH dose lowered serum triglyceride (TG) and nonesterified fatty acid (NEFA) levels and body fat. Both the high and medium doses also lowered serum leptin and insulin levels. Liver function testing revealed no adverse side effects under the current experimental conditions. The results of the present study suggest that LWDH has potential as a preventive or therapeutic natural product against overweight and obesity.

## 1. Introduction

The obesity epidemic is no longer limited to the developed countries but is now also rapidly spreading to developing countries [1, 2]. Associated with overweight and obesity is an increased risk for the development of cardiovascular disease (CVD), type 2 diabetes mellitus (T2DM), hypertension, dyslipidemia, and other complications [3]. The rapidly increasing prevalence of obesity has been negatively influencing the quality of human life and imposing an enormous burden on the health care system. Thus, control of body weight has now become one of the top priorities in nutritional and medical research. Studies have shown that a reduction of 5–10% body weight is sufficient to significantly improve many of the known obesity-related blood biochemistry parameters [4, 5], including hyperlipidemia, insulin and leptin resistance, and increased circulating nonesterified fatty acids (NEFA) [4–7]. 

Leptin and insulin play a critical role in energy homeostasis [8]. These two hormones can relay information about peripheral fat stores to the brain [9], by converging on specific neurons in the hypothalamus [10] and modulating eating behaviour and energy expenditure [11, 12]. One key feature of obesity is the development of insulin and leptin resistance [13, 14], resulting in an elevation of the circulating levels of these two hormones as a consequence of compensatory physiological responses to insulin and leptin insensitivity [13, 15]. With the onset of insulin and leptin resistance, changes occur in several other obesity-related blood biomarkers, such as an increase in NEFA and triglyceride (TG) levels in the circulation, as well as in the liver and muscle tissues [16]. 

To date, lifestyle changes, including dietary habits and exercise, remain the primary intervention for weight loss or control. However, long-term compliance has challenged its effectiveness. Different from other health problems and diseases, few dietary supplements or drugs have been successful in the prevention or treatment of obesity [17]. As a result, there exists a strong demand to continue searching for both safe and efficacious products to combat this emerging health epidemic. Traditional Chinese medicine (TCM) has been considered to have huge potential as an information source and starting point for the development of antiobesity products. The ancient TCM Liuwei Dihuang (LWDH), prepared from a basic formula of six Chinese herbs (*Radix Rehmanniae Preparata*, RRP; *Rhizoma Dioscoreae*, RD; *Fructus Corni*, FC; *Cortex Moutan*, CM; *Rhizoma Alismatis*, RA; *Poria*) [18, 19], is widely produced in China in accordance with the China Pharmacopoeia standard of quality control [20]. Despite its worldwide use for general health promotion [21], little is known about its potential benefits in regards to energy homeostasis and weight management. 

The aim of the present study was to evaluate the antiobesity effects of the Chinese herbal formulation, LWDH, by measuring body weight, food intake, body fat mass, blood lipid profiles, and the hormones leptin and insulin using a rat model of obesity. In order to evaluate potential toxicity, liver function testing was performed by analysing serum levels of the liver enzymes aspartate aminotransferase (AST), alanine aminotransferase (ALT), alkaline phosphatase (ALP), and *γ*-glutamyltransferase (GGT).

## 2. Materials and Methods

### 2.1. Animals and Diet

Sixty male CD rats were purchased from Charles River Laboratories (Montréal, Québec, Canada). Twelve of these animals were of the obesity-resistant strain (OR-CD), and 48 were of the obesity-prone strain (OP-CD). All animals were housed individually in cages in a temperature controlled room with a 12-hour light : dark cycle. Following a 2-week acclimation period with free access to regular rodent chow and water, the average body weight became 263 ± 21 g for the OR-CD rats and 300 ± 19 g for the OP-CD rats. The OR-CD rats served as the normal control group (OR), and the OP-CD rats were randomly divided into 4 groups (*n* = 12 each) prior to the commencement of treatment. One group of OP-CD rats served as the obesity control (OP), while the other 3 (T1A, T1B, and T1C) were treated for 10 weeks by twice daily oral gavage with total daily dosages of 500, 1500, or 3500 mg/kg BW of the concentrated LWDH pills (Wanxi Pharmaceuticals Co. Ltd., Henan, China) dissolved in water. The OR and OP control groups received water via oral gavage as the control vehicle. The gavage method was chosen versus adding to the diet in order to ensure dosage control. In addition to proper training, gavage feeding was optimized by using soft plastic gavage tubing, which produces much less stress than typical gavage needles. Treatment administration was performed during the light cycle, once in the morning and again 6 hours later. Throughout the treatment period, all rats were fed the AIN-93G diet modified to contain 60% energy from fat (lard : sunflower oil (96 : 4, wt/wt)). Body weight and food intake were recorded on a daily basis. At the end of the study, all animals were anaesthetized using Isoflurane (Abraxis BioScience, Richmond Hill, Ontario, Canada) following an overnight fast. Blood samples were collected via left ventricular cardiac puncture, placed on ice, and allowed to clot. After centrifugation, serum was collected, aliquoted, and stored at −80°C until analysis of each biomarker within a short period of time. Epididymal, perirenal, and omental fat depots were weighed and recorded, and the total body fat mass of the 3 portions was calculated. The animal use and experimental protocols were approved by the Joint Animal Care and Research Ethics Committee of the National Research Council Canada and the University of Prince Edward Island. The study was conducted in accordance with the guidelines of the Canadian Council on Animal Care.

### 2.2. Preparation and Dose Consideration of Concentrated LWDH Pills

The LWDH-concentrated pills used in the present study were manufactured by Henan Wanxi Pharmaceuticals Ltd. Co. (Nanyang, Henan, China) using the six Chinese herbs at a composition of 160 g RRP, 80 g RD, 80 g FC, 60 g CM, 60 g RA, and 60 g Poria. Briefly, following extraction with 95% ethanol, the residue of CM was mixed with all RRP, RA and Poria, and part of FC (27 g), followed by extraction with hot water (twice, 2 hr each). The water extract was then concentrated to form a paste and mixed with powdered RD and FC (53 g), and the ethanol extract of CM to form the final product. Based on the recommended daily human dosage of 24 pills (approximately 4.5 g/d), and the previously reported dosage of 2.4 g/kg/d in rats [22], 3 dosages of 500, 1500, or 3500 mg/kg BW were chosen in the present study.

### 2.3. Measurement of Serum Lipids

Lipid levels in the serum, including total cholesterol (T-C), TG, and HDL-cholesterol (HDL-C) were measured in duplicate using the TC Matrix Biochemistry Analyzer (Teco Diagnostics, Anaheim, Calif, USA) and supplied reagents. Non-HDL-C levels were calculated by subtracting HDL-C from T-C [23].

### 2.4. Measurement of Serum Nonesterified Fatty Acids

Serum NEFA levels were measured in duplicate using commercial kits (BioVision Research Products, Mountain View, Calif, USA), in accordance with the kit instructions. Standards were prepared at a series of concentrations and run in parallel with the samples. The levels of NEFA were calculated in reference to the corresponding standard curves and expressed as nmol/*μ*L.

### 2.5. Measurement of Serum Insulin and Leptin

Serum insulin and leptin levels were quantified in duplicate using commercial ELISA kits (Crystal Chem Inc., Downer's Grove, Ill, USA). Each assay was performed following the kit instructions. Standards at a series of concentrations were run in parallel with the samples. The insulin and leptin concentrations in the samples were calculated in reference to the corresponding standard curves and expressed as ng/mL.

### 2.6. Measurement of Liver Enzymes in the Serum

Liver enzyme levels in the serum, including AST, ALT, ALP, and GGT, were measured in duplicate using enzymatic methods on the TC Matrix Biochemistry Analyzer (Teco Diagnostics, Anaheim, Calif, USA) and supplied reagents.

### 2.7. Statistical Analysis

Data analyses were performed by one-way ANOVA using SAS 9.2 statistical software (SAS Institute, NC, USA). Differences between treatment means were determined by pair-wise comparisons using the least squares means test, with *P* < 0.05 indicating statistical significance. Results are presented as mean values with their standard errors.

## 3. Results

### 3.1. LWDH Significantly Lowered Body Weight in Obese Rats

**Figure 1:**
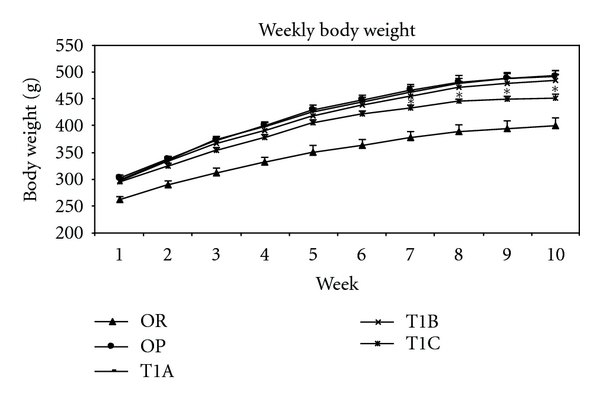
LWDH significantly lowered body weight in obese rats. OR: obesity-resistant normal control; OP: obesity-prone positive control; T1A, T1B, and T1C represent the 3 obesity-prone groups treated by twice daily oral gavage with total daily dosages of 500, 1500, or 3500 mg/kg BW of the LWDH herbal formulation, respectively. All animals were fed a high-fat diet (AIN-93G diet modified to contain 60% energy from fat (lard : sunflower oil (96 : 4, wt/wt)). Data were analyzed by one-way ANOVA. Differences between treatment means were determined by pair-wise comparisons using the least squares means test, with *P* < 0.05 indicating statistical significance. Results are presented as mean values with their standard errors (*n* = 12). *Differed significantly from OP (*P* < 0.05).

Throughout the 10-week treatment period, rats in the T1C group had significantly lower (*P* < 0.05) body weights compared to the obesity control OP rats (Figure 1). A significant body weight reduction in the TIC group was detected at week 7. This was further highlighted by a final body weight reduction of 8% compared to the OP rats, beginning at week 8 and persisting until the end of the study. The T1B group had a tendency towards reduced body weight but did not reach statistical significance, while the T1A group showed no effect.

### 3.2. Effect of LWDH on Body Fat Composition in Obese Rats

To determine the effect of LWDH on body fat mass and composition, epididymal, perirenal, and omental fat samples were excised and weighed. After the 10-week treatment period, rats in the T1C group showed significantly less (*P* < 0.05) fat mass in these regions compared to the OP control rats (Table 1). This observation was also noted in the total fat mass of the 3 portions.

### 3.3. Effect of LWDH on Weekly Food Intake in Obese Rats

During the first week of treatment, food intake was not affected. After 2 weeks, rats in the T1C group had reduced (*P* < 0.05) food intake compared to the OP controls, with this observation being noted until the end of the study (Table 2). Group T1B also showed a tendency towards decreased food intake, with significant reductions (*P* < 0.05) during weeks 3 and 4 compared to the OP control. Group T1A did not show any significant effect. A significant correlation was detected between food intake and body weight (*r* = 0.7094, *P* < 0.001).

### 3.4. Lipid-Lowering Effects of LWDH in Obese Rats

**Figure 2:**
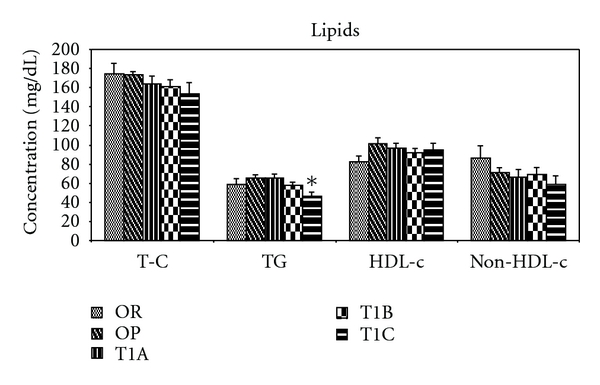
Lipid-lowering effects of LWDH in obese rats. OR: obesity-resistant normal control; OP: obesity-prone positive control; T1A, T1B, and T1C represent the 3 obesity-prone groups treated by twice daily oral gavage with total daily dosages of 500, 1500, or 3500 mg/kg BW of the LWDH herbal formulation, respectively. All animals were fed a high-fat diet (AIN-93G diet modified to contain 60% energy from fat (lard : sunflower oil (96 : 4, wt/wt)). Data were analyzed by one-way ANOVA. Differences between treatment means were determined by pair-wise comparisons using the least squares means test, with P < 0.05 indicating statistical significance. Results are presented as mean values with their standard errors (*n* = 12). *Differed significantly from OP (*P* < 0.05).

At the end of the study, the T1C group showed a 28% reduction (*P* < 0.05) in serum TG levels compared to the OP control. Serum T-C levels in the T1C group were reduced by 11% but did not reach statistical significance (*P* = 0.13) when compared to the OP control, due to large variations observed within the group (Figure 2). The levels of serum HDL-C and non-HDL-C in each of the LWDH treatment groups did not differ from the OP control.

### 3.5. LWDH Significantly Lowered Serum Nonesterified Fatty Acids in Obese Rats

**Figure 3:**
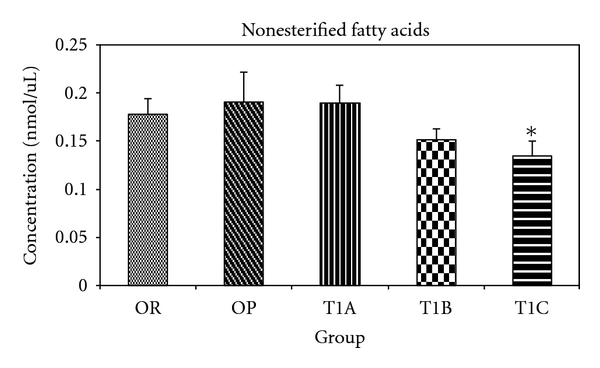
LWDH significantly lowered serum nonesterified fatty acids in obese rats. OR: obesity-resistant normal control; OP: obesity-prone positive control; T1A, T1B, and T1C represent the 3 obesity-prone groups treated by twice daily oral gavage with total daily dosages of 500, 1500, or 3500 mg/kg BW of the LWDH herbal formulation, respectively. All animals were fed a high-fat diet (AIN-93G diet modified to contain 60% energy from fat (lard : sunflower oil (96 : 4, wt/wt)). Data were analyzed by one-way ANOVA. Differences between treatment means were determined by pair-wise comparisons using the least squares means test, with *P* < 0.05 indicating statistical significance. Results are presented as mean values with their standard errors (*n* = 12). *Differed significantly from OP (*P* < 0.05).

Compared to the OP control, rats in the T1C group had significantly lower (*P* < 0.05) NEFA levels (Figure 3). In addition, the T1B group had marginally lower serum NEFA levels (*P* = 0.14) when compared to the OP control. The T1A group did not show any effect.

### 3.6. LWDH Significantly Lowered Serum Insulin and Leptin in Obese Rats

**Figure 4:**
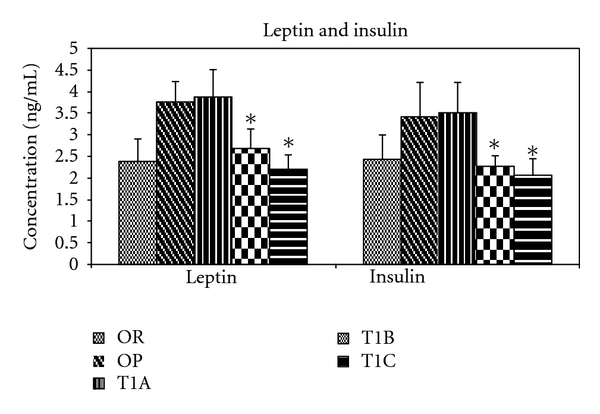
LWDH significantly lowered serum insulin and leptin in obese rats. OR: obesity-resistant normal control; OP: obesity-prone positive control; T1A, T1B, and T1C represent the 3 obesity-prone groups treated by twice daily oral gavage with total daily dosages of 500, 1500, or 3500 mg/kg BW of the LWDH herbal formulation, respectively. All animals were fed a high-fat diet (AIN-93G diet modified to contain 60% energy from fat (lard : sunflower oil (96 : 4, wt/wt)). Data were analyzed by one-way ANOVA. Differences between treatment means were determined by pair-wise comparisons using the least squares means test, with *P* < 0.05 indicating statistical significance. Results are presented as mean values with their standard errors (*n* = 12). *Differed significantly from OP (*P* < 0.05).

Serum levels of insulin and leptin were quantified at the end of the treatment period. In response to the T1B and T1C treatments, serum insulin and leptin levels were significantly reduced (*P* < 0.05) (Figure 4).

### 3.7. Serum Liver Enzyme Profile in Obese Rats in Response to LWDH Treatment

To assess the safety or potential toxicity of the LWDH concentrated pills, liver function tests were performed by measuring the serum levels of a suite of liver enzymes, including AST, ALT, ALP, and GGT. The results indicated that none of the 3 LWDH doses altered the serum levels of AST, ALT, or GGT (Table 3). Noteworthy, serum ALP levels were significantly reduced (*P* < 0.05) in the T1C group compared to the OP control.

## 4. Discussion

The results of the present study have highlighted the potential body weight-lowering capability of LWDH. When rats were treated with the high LWDH dose, body weights were lowered significantly after 7 weeks of treatment, and this effect was retained throughout the remainder of the treatment period. In line with the observed body weight reductions, epididymal, perirenal, and omental fat depots were also significantly lowered. The high LWDH dose also reduced serum TG and NEFA levels. Furthermore, the medium and high LWDH doses decreased serum insulin and leptin levels in a dose-dependent manner. These parameters are all highly related to obesity, and a reduction in any one is, in turn, beneficial to weight control and the mitigation of obesity-related complications [24–26].

Overweight and obesity have emerged and continue to grow as new threats to the quality of human life. Accumulating evidence, however, suggests that a modest weight reduction of 5–10% is sufficient to improve, or even abolish, several obesity-related complications [4, 27]. In the present study, LWDH reduced body weight in obese rats by over 8% after 7 weeks of treatment when provided at a dose of 3500 mg/kg/d. The 1500 mg/kg/d dose showed a tendency towards weight loss but did not reach a statistically significant level. A similar and dose-dependent reduction was observed in regards to food intake, which significantly correlated with weight changes, suggesting that the observed body weight reductions following LWDH treatment might, at least in part, be a result of decreased energy consumption. Food intake is influenced not only by nutritional status, but also by various palatability cues, including taste and smell [28, 29]. In the current study, LWDH was administered by oral gavage and thus should not have had any adverse effects on food taste. In addition, food intake was monitored on a daily basis throughout the entire study, and no significant decreases were observed throughout the first treatment week. If LWDH had an aversive taste, food intake would have been decreased immediately upon commencement of the treatment. As an aversion to taste was not seen, it is thought that LWDH might have inhibited appetite or induced satiety through interaction with the gut-brain axis, but this requires further investigation. Noteworthy is the fact that LWDH was administered in the form of a partially refined concentrated pill, which explains the high doses employed in the present study. The high dosage of LWDH was similar to a diabetes study in rats [22] and higher than the human dosage used for general health promotion [30]. The dosage for weight management in humans has not yet been documented. The current dosage can be significantly reduced when administered in the form of an extract or fraction and following dosage optimization. Furthermore, with respect to translation into the potential for human use, thrice daily dosing is recommended in TCM for general health promotion. Studies have shown twice daily oral dosing regimes to be equal [31] or superior [32] to once daily regimes in terms of efficacy and safety. 

Hyperlipidemia and increased circulating free fatty acids are generally associated with obesity [26]. In the present study, the high LWDH dose significantly lowered blood TG and NEFA levels, in line with the observed body weight reductions, and showed a tendency towards lowering T-C levels. It is well known that in addition to elevated circulating cholesterol levels, increased blood TG levels are an independent risk factor for the development of atherosclerosis and CVD [25]. Metabolically, fatty acids and TG are interrelated, and their blood levels change in proportion to one another [33]. Elevated circulating levels of TG and NEFA are commonly observed in obese individuals, resulting from increased fat mobilization from adipose tissues [34, 35], and are further elevated with the onset of insulin and/or leptin resistance [36]. Improvement of insulin and/or leptin sensitivity stimulates fatty acid oxidation, resulting in a reduction in circulating TG and NEFA levels [37]. The reductions in circulating TG and NEFA levels might be a consequence of LWDH-mediated body weight reduction, resulting in improved insulin and leptin sensitivity and thus fatty acid oxidation, in addition to reduced fat mobilization from adipose tissues and a consequential decrease in circulating NEFA levels following weight loss.

Energy homeostasis requires a highly integrated neurohumoral control system involving several hormones, including leptin and insulin [37, 38]. Leptin and insulin are secreted in direct proportion to adipose tissue mass [37]. In the obese state, an increase in adipose tissue mass leads to increased secretion of insulin and leptin into the circulation [38], often resulting in the development of hyperinsulinemia and hyperleptinemia, respectively [39, 40]. Normally, these hormones can relay information about peripheral fat stores to the brain and effectively modulate food intake and energy expenditure [12, 41], through inhibition and activation of anabolic and catabolic pathways, respectively [39]. However, in the obese state, insensitivity to the antiobesity effects of these regulatory hormones often develops [13, 14]. In the present study, the medium and high LWDH doses significantly reduced blood levels of insulin and leptin, which were correlated with the observed weight changes, especially in response to the high dose. The reduced levels of both hormones in the serum could be a result of the observed weight reduction by LWDH and subsequent improvements of their sensitivity. Nevertheless, it is not possible to exclude the prospect that LWDH also directly affected insulin and leptin sensitivity, independent of weight changes. 

As LWDH is an herbal formulation, it was also of interest to determine if any toxic effects may be linked to the application of this product. Rats were monitored on a daily basis, and no abnormal behaviours or activities were noted throughout the entire study period. In addition, liver function tests which are used routinely to detect the potential toxicity of a product [42] were conducted. Analysis of 4 liver enzymes that are generally used in clinical testing did not reveal any toxic effect of LWDH under the current experimental conditions. Furthermore, blood concentrations of liver enzymes are indicative of a tendency towards the development of obesity [43]. Decreased concentrations of these enzymes in obese individuals reflect improved insulin sensitivity and reduced fat accumulation in the liver [43], whereas elevated levels represent an increased risk for the development of T2DM and CVD [44, 45]. The observed reductions in serum ALP levels in response to the high LWDH dose might be indicative of improved insulin sensitivity, in line with the significantly reduced blood insulin and leptin levels, in addition to the potential improvement of liver function following weight loss. 

In conclusion, the present study has demonstrated the antiobesity effects of LWDH in obese rats, without any observable toxic effects as evaluated by liver function testing. Moreover, LWDH improved blood lipid profiles, including NEFA, and lowered blood leptin and insulin levels, which may be indicative of an improvement in insulin and leptin sensitivity. The results presented herein have shed light on the potential for the development of LWDH as a natural product for the management of obesity and its related complications. These findings could be related to the regulation of LWDH on appetite, which awaits further investigation.

## Figures and Tables

**Table 1:** Effect of LWDH on body fat composition in obese rats^†^ (mean values with their standard errors, *n* = 12).

	Control groups				LWDH-treated groups					
	OR		OP		T1A		T1B		T1C	
	Mean	SE	Mean	SE	Mean	SE	Mean	SE	Mean	SE
Epididymal (g)	5.54	0.29	9.70	0.75	9.60	0.65	8.56	0.48	6.96*	0.51
Perirenal (g)	6.79	1.32	11.00	1.39	10.62	1.23	8.84	0.80	7.91*	0.79
Omental (g)	7.71	1.29	11.21	0.85	12.46	0.81	10.64	0.43	8.72*	0.67

Total (g)	19.58	2.16	31.92	2.49	32.67	2.33	28.03	1.43	23.59*	1.68

LWDH: Liuwei Dihuang; OR: obesity-resistant normal control group; OP: obesity-prone positive control group; T1A: 500 mg/kg/d LWDH group; T1B: 1500 mg/kg/d LWDH group; T1C: 3500 mg/kg/d L WDH group.

*Mean values within a row were significantly different from OP (pair-wise comparisons using the least squares means test; *P* < 0.05).

^†^The change in body fat mass in obese rats treated with total daily dosages of 500, 1500, or 3500 mg/kg BW LWDH is given.

**Table 2:** Effect of LWDH on weekly food intake in obese rats^†^ (mean values with their standard errors, *n* = 12).

	Control groups				LWDH-treated groups					
	OR		OP		T1A		T1B		T1C	
	Mean	SE	Mean	SE	Mean	SE	Mean	SE	Mean	SE
Week 1 (g)	13.88	0.79	14.57	0.62	15.42	0.62	15.39	0.44	15.17	0.40
Week 2 (g)	14.43	0.61	15.34	1.88	15.13	0.36	14.14	0.35	13.08*	0.61
Week 3 (g)	12.91	0.63	14.41	0.55	15.07	0.44	12.55*	0.54	11.92*	0.52
Week 4 (g)	13.35	0.71	15.68	0.89	15.03	0.51	13.68*	0.62	13.11*	0.36
Week 5 (g)	13.67	0.49	17.07	0.38	16.31	0.55	15.11	0.89	15.93*	0.54
Week 6 (g)	14.39	0.64	14.36	0.64	14.31	0.73	13.75	0.45	13.40*	0.42
Week 7 (g)	14.76	0.65	15.81	0.78	15.78	0.96	14.68	0.85	13.58*	0.51
Week 8 (g)	12.46	0.86	14.16	0.90	13.59	0.52	13.14	0.51	12.29*	0.51
Week 9 (g)	12.86	1.02	13.68	0.65	14.48	0.64	13.12	0.76	13.25*	0.63
Week 10 (g)	12.96	0.84	14.42	0.73	14.15	0.58	15.10	0.77	13.72*	0.65

LWDH: Liuwei Dihuang; OR: obesity-resistant normal control group; OP: obesity-prone positive control group; T1A: 500 mg/kg/d LWDH group; T1B: 1500 mg/kg/d LWDH group; T1C: 3500 mg/kg/d LWDH group.

*Mean values within a row were significantly different from OP (pair-wise comparisons using the least squares means test; *P* < 0.05).

^†^The change in weekly food intake in obese rats treated with total daily dosages of 500, 1500, or 3500 mg/kg BW LWDH is given.

**Table 3:** Serum liver enzyme profile in obese rats in response to LWDH treatment^†^ (mean values with their standard errors, *n* = 12).

	Control groups				LWDH-treated groups					
	OR		OP		T1A		T1B		T1C	
	Mean	SE	Mean	SE	Mean	SE	Mean	SE	Mean	SE
AST (U/L)	197.54	25.14	178.79	9.67	180.46	12.09	187.25	13.53	199.46	12.77
ALT (U/L)	38.00	1.83	46.04	1.95	44.88	1.51	41.88	1.16	44.45	2.06
ALP (U/L)	168.21	13.15	295.75	1.69	269.38	6.16	271.29	15.58	252.63*	11.68
GGT (U/L)	8.71	0.14	9.12	0.25	8.83	0.33	9.25	0.23	8.77	0.42

LWDH: Liuwei Dihuang; OR: obesity-resistant normal control group; OP: obesity-prone positive control group; T1A: 500 mg/kg/d LWDH group; T1B: 1500 mg/kg/d LWDH group; T1C: 3500 mg/kg/d LWDH group.

*Mean values within a row were significantly different from OP (pair-wise comparisons using the least squares means test; *P* < 0.05).

^†^The change in serum liver enzyme profiles in obese rats treated with total daily dosages of 500, 1500, or 3500 mg/kg BW LWDH is given.
